# Differences in Alzheimer's disease blood biomarker stability: Implications for the use of tau/amyloid ratios

**DOI:** 10.1002/alz.70173

**Published:** 2025-05-01

**Authors:** Daniel J. Figdore, Bethany J. Schuder, Susan Ashrafzadeh‐Kian, Tina Gronquist, Joshua A. Bornhorst, Alicia Algeciras‐Schimnich

**Affiliations:** ^1^ Department of Laboratory Medicine and Pathology Mayo Clinic Rochester Minnesota USA

**Keywords:** Alzheimer's disease, amyloid beta, amyloid pathology, blood‐based biomarkers, immunoassays, plasma phosphorylated tau217, pre‐analytic, stability

## Abstract

**INTRODUCTION:**

We compare the stability of phosphorylated tau (p‐tau)217, amyloid beta (Aβ)42, Aβ40, Aβ42/40, and p‐tau217/Aβ42 at different storage temperatures.

**METHODS:**

Ten ethylenediaminetetraacetic acid–plasma sample aliquots stored at frozen, refrigerated, and ambient temperatures were tested at various timepoints up to 30 days. The mean percent change from baseline was calculated.

**RESULTS:**

Aβ42 and Aβ40 concentrations decreased by >15% after 6 hours at ambient temperature and after 24 hours refrigerated, while p‐tau217 remained stable over 72 hours with < 10% deviation from baseline at either storage temperature. The Aβ42/40 ratio remained relatively constant as each analyte concentration decreased concurrently, while the p‐tau217/Aβ42 ratio deviated from baseline over time. All biomarkers were stable up to 30 days frozen.

**DISCUSSION:**

Differences in the stability of Aβ42 and p‐tau217 may lead to altered p‐tau217/Aβ42 ratio results if samples are not handled properly during the pre‐analytical testing phase. Ideally, samples should be frozen promptly and sent to the laboratory frozen.

**Highlights:**

- Plasma phosphorylated tau (p‐tau)217, amyloid beta (Aβ)42, Aβ40, Aβ42/40, and p‐tau217/Aβ42 stability was assessed.
- P‐tau217 was stable, but Aβ42 and Aβ40 started decreasing by 6 hours at ambient temperature.
- The analyte stability differences led to falsely increased p‐tau217/Aβ42 ratios.
- Increases > 15% were seen in p‐tau217/Aβ42 after 6 hours at ambient temperature or 24 hours at 4°C.

## 1 BACKGROUND

Alzheimer's disease (AD) is the most common cause of dementia,^1^ characterized by the presence of amyloid beta (Aβ) plaques and intracellular neurofibrillary tangles containing hyperphosphorylated tau (p‐tau) species.^2^, ^3^ Recently developed disease‐modifying therapies targeting Aβ accumulation have increased the demand for widely accessible and non‐invasive methods of detecting amyloid pathology.^4^ Currently, the most well‐characterized available blood biomarkers (BBMs) for detection of amyloid pathology are p‐tau217, p‐tau181, and the Aβ42/40 ratio, with assays incorporating p‐tau217 being the most diagnostically accurate BBM.^5^ More recently, the use of the plasma p‐tau217/Aβ42 ratio has been suggested for the detection of amyloid pathology, with purportedly modestly higher performance than p‐tau217 alone.^6^, ^7^, ^8^, ^9^

A number of studies using either immunoassay or liquid chromatography with tandem mass spectrometry (LC‐MS‐MS) p‐tau217 assays have reported a high diagnostic accuracy of the p‐tau217 BBM for the detection of amyloid pathology with receiver operating characteristic (ROC) curves of ≥ 0.90, and comparable, in some cases, to the performance of cerebrospinal fluid (CSF) AD biomarkers.^10^, ^11^, ^12^ The 2024 revised criteria for diagnosis and staging of AD considers the use of p‐tau217 assays—with a diagnostic accuracy of 90% for the identification of amyloid pathology—an acceptable BBM for the diagnosis of AD.^13^ Plasma p‐tau217 tests are now being increasingly incorporated into clinical practice for the detection of amyloid pathology.^4^, ^6^, ^14^ However, successful widespread adoption of BBMs requires further understanding of both analytical and pre‐analytical factors that might influence the interpretation of these biomarkers which would allow for the development of standardized sample collection protocols.

Pre‐analytic stability of biomarkers is known to impact laboratory tests and efforts have been made to standardize pre‐analytic variables for AD BBMs in research.^15^ In clinical practice, pre‐analytic factors like stability can lead to inaccurate results that may result in incorrect clinical decisions.^16^ Here, we aimed to assess the differences in analyte stability of p‐tau217, Aβ42, and Aβ40 as well as how these pre‐analytic differences affect the p‐tau217/Aβ42 and Aβ42/40 ratios within the same cohort.

## 2 METHODS

### 2.1 Participants and design

A total of 10 specimens were included for each analyte at each storage temperature condition. Samples were obtained from two sources. Participants presenting with symptoms of cognitive decline (cognitively impaired group) were recruited to donate an ethylenediaminetetraacetic acid (EDTA)–plasma sample if they were undergoing either amyloid positron emission tomography (PET) imaging or CSF AD biomarker testing as part of their clinical diagnosis at Mayo Clinic, Rochester, Minnesota. Fourteen of these participants with confirmed amyloid pathology who had recent plasma collections were chosen randomly to include in this study. Specimens from 10 of these individuals were included in the ambient and refrigerated studies for all analytes, and four were included in the frozen p‐tau217 study. Additionally, 16 freshly collected samples were obtained from healthy volunteers. Specimens from 10 of these individuals were included in the frozen studies for Aβ42 and Aβ40, and 6 were included in the frozen p‐tau217 study. Written informed consent was obtained from all who participated.

**RESEARCH IN CONTEXT**

1. **Systematic review**: The authors reviewed literature in PubMed related to the pre‐analytic properties of plasma phosphorylated tau (p‐tau)217, amyloid beta (Aβ)42, Aβ40, Aβ42/40, and p‐tau217/Aβ42. While a limited number of publications were identified, none so far had examined the impact of the analyte stability on the p‐tau217/Aβ42 ratio.
2. **Interpretation**: Our findings show that there are significant differences in the stability of p‐tau217, Aβ42, and Aβ40 when samples are stored at ambient and refrigerated temperatures. While p‐tau217 is stable up to 72 hours at either temperature, Aβ42 and Aβ40 concentrations start decreasing by > 15% after 6 hours at ambient temperature or 24 hours refrigerated. Differences in the stability of Aβ42 and p‐tau217 led to altered p‐tau217/Aβ42 ratio results.
3. **Future directions**: The information provided here could help laboratories create pre‐analytic protocols for the collection of these Alzheimer's disease biomarkers in clinical practice and prevent inaccurate p‐tau217/Aβ42 ratio results.

### 2.2 Plasma collection and handling

EDTA–plasma samples collected from the cognitively impaired group were immediately centrifuged and plasma aliquoted into polypropylene tubes and stored frozen at −80°C. Within ≤ 7 days from collection, aliquoted samples were thawed, and biomarker concentrations were measured (baseline). Thereafter, aliquots were stored at various temperatures and measured at the predefined timepoints. EDTA–plasma samples from the healthy volunteers were processed immediately after collection. Plasma was separated and aliquoted into polypropylene tubes before measuring baseline concentrations, then aliquots were stored at the appropriate temperatures and measured at the predefined timepoints.[Fig alz70173-fig-0001]

Separate aliquots of samples were kept frozen at −20°C, refrigerated at 4°C, and at ambient temperature (23°C) throughout the duration of the study. Directly prior to analysis at each timepoint, specimens were brought to room temperature (if applicable), vortexed to mix, and centrifuged at 4000 × g for 5 minutes. Immediately after testing, specimens were returned to their appropriate storage temperatures.

### 2.3 Biomarker immunoassay testing

Testing was performed using the Fujirebio Lumipulse G1200 fully automated chemiluminescent enzyme immunoassay analyzer with the Lumipulse G p‐Tau 217 Plasma (catalog number: 81472, lot number: 4129), Lumipulse G β‐Amyloid 1‐42 Plasma (catalog number: 81301, lot number: 5081), and Lumipulse G β‐Amyloid 1‐40 Plasma (catalog number: 81298, lot number: 5081) kits. All results were expressed in pg/mL. Manufacturer quality control materials were tested each day and assessed to be within the manufacturer's listed ranges prior to sample testing. The following timepoints were evaluated for all analytes: refrigerated (4°C) and ambient (23°C) temperatures: 6, 12, 24, and 72 hours; frozen (−20°C): 1, 7, 14, and 30 days.

### 2.4 Data analysis

All data were analyzed by calculating the percent change at each timepoint compared to the baseline timepoint zero concentration as ([(timepoint − baseline)/baseline] × 100%). The mean percent change at each timepoint was calculated, with a > ± 15% change from baseline considered significant. The mean percent change was plotted versus time. All data analysis was performed, and figures were created using Microsoft Excel version 2412.

## 3 RESULTS

**FIGURE 1 alz70173-fig-0001:**
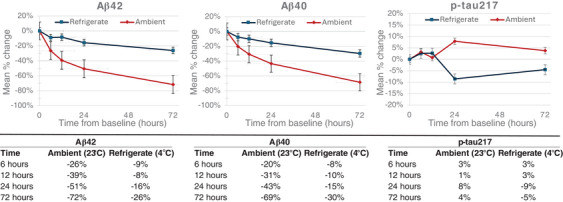
Stability of p‐tau217, Aβ42, and Aβ40 at refrigerated and ambient temperatures. Mean percent change from baseline at 6, 12, 24, and 72 hours. Aβ, amyloid beta; p‐tau, phosphorylated tau

**FIGURE 2:**
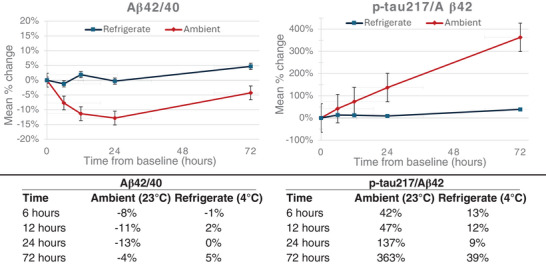
Stability of the p‐tau217/Aβ42 and the Aβ42/Aβ40 ratios at refrigerated and ambient temperatures. Mean percent change from baseline at 6, 12, 24, and 72 hours. Aβ, amyloid beta; p‐tau, phosphorylated tau

**FIGURE 3:**
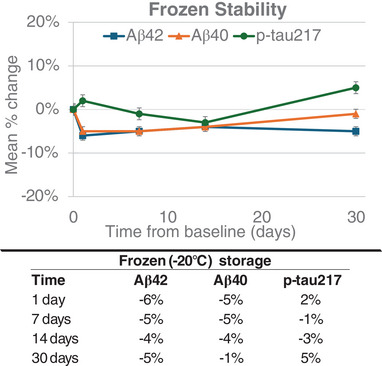
Frozen (−80°C) stability of p‐tau217, Aβ42, and Aβ40. Mean percent change from baseline at 1, 12, 24, and 72 hours. Aβ, amyloid beta; p‐tau, phosphorylated tau

Mean percent change is plotted versus time to show potential deviation from the baseline of each biomarker stored at the indicated ambient and refrigerated conditions for p‐tau217, Aβ42, and Aβ40 (Figure 1), as well as the p‐tau217/Aβ42 and the Aβ42/40 ratios (Figure 2). Significant mean percent changes from baseline concentrations (> ± 15%) for Aβ42 and Aβ40 were observed at both ambient and refrigerated temperatures after 6 hours, and 24 hours, respectively. Similarly, the p‐tau217/Aβ42 ratio displayed significant deviation (increase) from the baseline timepoint at ambient temperature after 6 hours, due to the greater degradation rates observed for Aβ42 compared to p‐tau217 at ambient temperature. A similar effect of smaller magnitude was observed at refrigerated temperature, with the p‐tau217/Aβ42 ratio deviating by > 15% at 72 hours. In contrast, the Aβ42/40 ratio and p‐tau217 showed little variability across the timepoints compared to the baseline at all storage temperature conditions. All analytes remained stable while frozen for up to 30 days with ≤ 5% deviation from baseline concentration, as shown in Figure 3.

## 4 DISCUSSION

These data support other findings that ambient or refrigerated plasma Aβ42 and Aβ40 concentrations decrease significantly after 6 hours, but that the concordant decrease does not significantly impact the resulting Aβ42/40 ratio.^17^, ^18^ This study also supports previous findings showing good stability of ambient or refrigerated plasma p‐tau217.^19^, ^20^ Frozen (−20°C) concentrations were shown to be stable, as all of the analytes deviated < 6% from baseline at all timepoints. This minimal variation indicates good frozen stability over at least 30 days. However, while ambient or refrigerated stability had little impact on Aβ42/40, it did significantly impact the p‐tau217/Aβ42 ratio. Prolonged sample storage at ambient (≥ 6 hours) or refrigerated (≥ 24 hours) conditions caused elevation of p‐tau217/Aβ42 in one of two negative baseline samples which resulted in a false‐positive result based on a predefined cutpoint of > 0.0075.^6^ When using p‐tau217/Aβ42 for research or clinical decision making, care must be taken to ensure samples are frozen immediately if testing will not be completed within 6 hours of collection.

The use of a two‐cutpoint approach has been described for p‐tau217.^3^, ^6^, ^21^ The use of this approach for the p‐tau217/Aβ42 ratio test could help to decrease the potential of false positive results related to analyte stability differences by classifying slightly falsely elevated results from a negative to an intermediate, which would trigger follow‐up testing instead of classifying the results as positive for amyloid pathology.

A limitation of this study is that not all samples were measured fresh, as a subset was measured after one freeze/thaw cycle. This was due to the need to have samples with higher concentrations of p‐tau217, because in healthy donors p‐tau217 concentrations are near the lower limit of quantitation of the assay. This was overcome by measuring previously frozen samples from individuals presenting with cognitive decline and having a positive amyloid PET or CSF AD biomarker result, which gave higher p‐tau217 concentrations. The use of samples after one freeze/thaw cycle was considered acceptable as previous literature^19^, ^20^, ^22^, ^23^ has shown freeze/thaw cycles do not significantly impact concentrations of these analytes in plasma.

In conclusion, all analytes showed excellent pre‐analytic stability under frozen storage conditions. Plasma p‐tau217 exhibited good pre‐analytic stability at refrigerated and ambient temperatures, while Aβ42 and Aβ40 concentrations decreased rapidly under these storage conditions. The Aβ42/Aβ40 ratio was minimally affected by the concurrent decrease in Aβ42 and Aβ40 concentrations. However, the p‐tau217/Aβ42 ratio increased significantly with the decrease of Aβ42 concentration. Implementation of a p‐tau217/Aβ42 ratio test in the clinical setting must consider if the modest increase in the clinical performance compared to p‐tau217 outweighs the risk of potential false positive results if the sample is not frozen promptly after collection.

## CONFLICT OF INTEREST STATEMENT

Alicia Algeciras‐Schimnich has participated on advisory boards for Roche Diagnostics and Fujirebio Diagnostics and received an honorarium from Roche Diagnostics. Joshua A. Bornhorst has participated and received an honorarium from Roche Diagnostics. Daniel J. Figdore, Bethany J. Schuder, Susan Ashrafzadeh‐Kian, and Tina Gronquist report no disclosures. Author disclosures are available in the supporting information.

## CONSENT STATEMENT

All human subjects provided informed consent.

## Supporting information



Supporting Information
